# Active Immunization in Tuberculosis

**Published:** 1912-02

**Authors:** 


ACTIVE IMMUNIZATION IN TUBERCULOSIS, Dr. H. S. Achard of Chicago, *Illinois Medical Journal,* August 1911. Passive immunization (i.e. by injection of sera obtained from animals infected with virulent bacilli, as by Marmorek's and Maragliano's methods) has failed. Active immunization (by direct injection of germ products into the patient) has yielded good results. He cites the following statistics:

| Authority | General Treatment | | | Active Immunization | | |
|---|---|---|---|---|---|---|
| | No. of cases | per ct. cured | per ct. relieved | No. of cases | per ct. cured | per ct. relieved |
| Langenbach & Weil | 99 | 9 | 45 | 99 | 42 | 40 |
| Pottenger (collected from literature, first stage) cases) | 611 | 64 | | 496 | 84 | |
| von Ruck at Winyah | 782 | 11.9 | 30.5 | | | |
| von Ruck at Winyah tuberculin | | | | 723 | 36.8 | 42.8 |
| Watery Ext. of T. B. | | | | 1503 | 55.5 | 33.8 |
| Collected from literature, watery ext. of T. B. | | | | 2183 | 50.3 | 29.4 |

Von Ruck also found that 90 per cent. of cases apparently cured by general treatment, relapsed and only 20 per cent. of those apparently cured by active immunization. Achard also discusses technic quite thoroughly.

